# The impact of CFTR modulator triple therapy on type 2 inflammatory response in patients with cystic fibrosis

**DOI:** 10.1186/s13223-023-00822-2

**Published:** 2023-07-31

**Authors:** A. M. Mehta, I. Lee, G. Li, M. K. Jones, L. Hanson, K. Lonabaugh, R. List, L. Borish, D. P. Albon

**Affiliations:** 1 University of Virginia School of Medicine, Charlottesville, VA USA; 2 Department of Medicine, Baylor College of Medicine, Houston, TX USA; 3 Department of Statistics, University of Virginia, Charlottesville, VA USA; 4 Public Health Sciences, University of Virginia, Charlottesville, VA USA; 5 Department of Medicine, University of Virginia, Charlottesville, VA USA; 6 Department of Microbiology, University of Virginia, Charlottesville, USA; 7 Departments of Medicine, University of Virginia School of Medicine, 800546, Charlottesville, VA 22908 USA

## Abstract

**Background:**

Treatment of cystic fibrosis (CF) has been revolutionized by the use of cystic fibrosis transmembrane conductance regulator (CFTR) protein modulators such as elexacaftor/tezacaftor/ivacaftor (ETI) triple therapy. Prior studies support a role for type 2 (T2) inflammation in many people with CF (PwCF) and CF-asthma overlap syndrome (CFAOS) is considered a separate clinical entity. It is unknown whether initiation of ETI therapy impacts T2 inflammation in PwCF. We hypothesized that ETI initiation decreases T2 inflammation in PwCF.

**Methods:**

A single center retrospective chart review was conducted for adult PwCF. As markers of T2 inflammation, absolute eosinophil count (AEC) and total immunoglobulin E (IgE) data were collected longitudinally 12 months prior to ETI therapy initiation and 12 months following therapy initiation. Multivariable analyses adjusted for the age, gender, CFTR mutation, disease severity, inhaled steroid use, and microbiological colonization.

**Results:**

There was a statistically significant reduction (20.10%, p < 0.001) in 12-month mean total IgE following ETI initiation; this change remained statistically significant in the multivariate model. The longitudinal analysis demonstrated no change in AEC following therapy initiation.

**Conclusion:**

This study demonstrates that there is a statistically significant percent reduction in mean total IgE but no change in AEC following ETI initiation. ETI may lead to decreased antigen and superantigen load in the airway as a result of improved mucociliary clearance and these changes may drive the decline in total IgE, without influencing the epigenetic drivers of eosinophilic inflammation. Further studies are warranted to determine the underlying mechanism of ETI impact on T2 inflammation and possible role for asthma immunomodulator therapy post ETI initiation in CFAOS.

Cystic fibrosis (CF) is one of the most common respiratory genetic diseases, affecting an estimated ~ 37,000 people in North America and ~ 162,000 people worldwide [1]. It is characterized by mutations in the *CFTR* gene which encodes the cystic fibrosis transmembrane conductance regulator (CFTR) protein, most commonly a deleted phenylalanine in the 508th position (F508del). CFTR is a chloride channel that regulates transport of sodium, chloride, and bicarbonate [2]. Classic pulmonary symptoms of CF include thick, mucous secretions in the airway causing chronic pulmonary infection, increase bacterial load and fungal colonization, and obstructive lung symptoms, which progress to airway edema, mucus plugging with airway collapse, and bronchiectasis [2].

Many studies have shown that CF patients commonly display evidence of type 2 (T2) inflammation [3] characterized by evidence of airway and peripheral eosinophilia, as well as high levels of total and allergen-specific immunoglobulin E (IgE) [3, 4]. T2 inflammation is classically implicated in the pathogenesis of allergic as well as non-allergic eosinophilic subtypes of asthma [3, 5]. But can also develop in other lung inflammatory disorders including, for example, being highly prevalent in eosinophilic chronic obstruction pulmonary disease. T2 inflammation represents, in large part, the response of the airway epithelial cells to airspace inflammation with the consequent production of numerous cytokines (including the alarmins thymic stromal lymphopoietin (TSLP), interleukin (IL)-25, and IL-33) and chemokines that are associated with promoting the T2 inflammation signature. These mediators both directly drive the generation of the classic T2 cytokines IL-4, IL-5, and IL-13 from innate lymphoid 2 cells (ILC2s), mast cells, and others but are also responsible for the differentiation of the antigen-specific Th2 effector cells that also produce these cytokines [3, 4], Loss of epithelial barriers allow for allergens and microbes to access stromal tissue and further promote the activation of Th2 effector lymphocytes and B cells [5]. CF patients may be additionally prone to T2 inflammation due to the increased fungus colonization and infection in their airways [6]. T2 inflammation may have importance in tissue repair following airway injury, and antimicrobial responses [3, 4]. In addition, there is evidence that dysfunction of CFTR on T lymphocytes may promote Th2 immune deviation [7–10].

Several studies support this role for T2 inflammation in many patients with CF. Siedlecki, *et. al.* found a prevalence of peripheral eosinophilia of 45% in CF patients hospitalized for exacerbation, and eosinophilia was associated with significantly longer hospital stays [11]. Our group previously found a correlation between T2 inflammation, based on peripheral eosinophilia and total IgE, frequent exacerbations and lung function decline, based on peripheral eosinophilia and IgE levels and we have reported on the ability of eosinophil-targeting therapeutics to produce beneficial outcomes in CF patients [12, 13]. Interestingly, according to the 2021 CF Foundation Patient Registry Annual Data Report, the prevalence of asthma in CF patients is 30.8% [14]. Experts in the CF community have acknowledged the association between asthma and CF, now considering CF-asthma overlap syndrome (CFAOS) a separate clinical entity [15]. Previous studies support this concept that elevations in type 2 inflammatory markers likely reflect an intrinsic eosinophilic phenotype of CF [4, 5].

Elexacaftor/tezacaftor/ivacaftor (ETI) modulator therapy has recently been approved by Food and Drug Administration and has become the main therapy prescribed for most people with CF. ETI has been shown to improve CFTR function, decrease sweat chloride concentrations, and improve both lung function and functional status as demonstrated in the PROMISE clinical trial. [16]. While addressing the defect in CFTR function, it is unknown whether these agents impact the presence of a previously established T2 inflammatory state that developed as a complication of CF inflammation.

The aim of this study was to investigate the impact of CFTR modulator triple therapy on Absolute Eosinophil Count (AEC) and total IgE as markers of the type 2 inflammatory response in patients with CF.

## Methods

### Patient population

A single center retrospective chart review was conducted for adult cystic fibrosis patients (n = 108) seen at the University of Virginia Adult Cystic Fibrosis Clinic between 2018 and 2021. Inclusion criteria were a diagnosis of CF and initiation of ETI therapy and at least one measurement of IgE and/or AEC pre and post ETI initiation. Exclusion criteria were a history of transplant, IL-4 (dupilumab), IL-5 (benralizumab, mepolizumab) or IgE (omalizumab)-targeting biologics, and insufficient AEC or total IgE data. This study was approved by our institution’s Institutional Review Board Human Subjects Research committee and followed procedures with ethical standards. (IRB-HSR 23337).

### Data collection

AEC and IgE data were collected for each patient 12 months prior to initiation of ETI therapy and 12 months following initiation of therapy. AEC measurements from the center-associated lab were collected from the electronic health record (EHR). If more than one IgE or AEC measurements were available, the mean pre and post-ETI levels were calculated and included in the analysis. Given that infection results in activation of inflammatory responses, we excluded data collected during pulmonary exacerbation and/or use of IV antibiotics. AEC measurements were excluded if they were obtained within 7 days prior to determination of an exacerbation and/or initiation of IV antibiotic therapy or if they were within 14 days following exacerbation and/or completion of IV antibiotic therapy.

We were unable to exclude total IgE measurements taken during an exacerbation as total IgE measurements were obtained on a less frequent basis, often only once annually. As such, total IgE data were collected from the EHR including both data from the center associated lab and outside facilities, when available. Additional collected data included age, sex, disease severity, infection/colonization history, CFTR mutation information, and inhaled steroid use. Disease severity was defined based on percent predicted Forced Expiratory Volume in one second (ppFEV_1_) and was categorized as normal (ppFEV_1_ > 90%), mild (70% < ppFEV_1_ ≤ 90%), moderate (40% < ppFEV_1_ ≤ 70%), and severe (ppFEV_1_ ≤ 40%). With regards to bacterial colonization history, data regarding prior *Pseudomonas* infection, MRSA infection, and other bacterial colonization were collected. Other bacteria were defined as *Stenotrophomonas* spp., *Achromobacter* spp., *Acinetobacter* spp., or *Burkholderia* spp. With regards to fungal colonization, data regarding prior *Aspergillus* spp., *Exophiala* spp., and *Rasamsonia* spp. colonization were collected. Colonization was defined as 2 positive cultures in a 6 months interval. The bacterial and fungal colonization was determined at the beginning of the study, for the following reasons: after ETI initiation and in the context of the pandemic, sputum collection declined; the program transitioned to telemedicine and remote sputum collection was challenging; in addition, the patient experienced a significant decrease in sputum production due to ETI. Inhaled steroid use was coded as yes or no, defined as use prior to ETI initiation.

### Statistical analysis

All statistical analyses were performed in R (version 4.2.2). A two-sided significance level of 0.05 was selected as the null hypothesis rejection criterion. Linear mixed-effect models were fit using the lme4 [17] and lmerTest [18] packages. In multivariable models, all clinically relevant covariates were chosen a priori. Before performing inferences on any regression model, residual diagnostics were checked to verify assumptions of normality and homoskedasticity. The responses, AEC and mean total IgE, were natural log transformed to induce normality and correct heteroskedasticity. Due to the log transformation, coefficients are interpreted as percent changes rather than absolute changes in the response. Inferences on model coefficients and contrasts were conducted with t-test using the emmeans package [19] and the F-test.

To assess how log-transformed 12-month mean total IgE changed after ETI initiation, we fit a multivariable linear mixed-effect model with a random intercept of patient, regressing over an indicator variable for ETI initiation and adjusting for current age, gender, CFTR mutation, initial disease severity level, *Pseudomonas* spp. colonization, MRSA colonization, other bacterial colonization, fungal colonization, and inhaled steroid use. A linear mixed-effect model was constructed to further investigate the mediating effect of initial disease severity on the ETI treatment effect. The model treated initial disease severity as a factor with four levels.

The relationships between the change in mean total IgE after ETI and prior fungal colonization, *Pseudomonas* colonization, or MRSA colonization were each examined individually with two linear mixed-effect models (a univariate and multivariable model with covariates age, gender, CFTR mutation, initial disease severity level, and inhaled steroid use) that had a random intercept for patient. All models included an interaction between the presence of the pathogen and ETI initiation.

To assess how log-transformed AEC changed after ETI initiation, we fit two linear mixed-effect models, a univariate and multivariable model, with a random intercept for patient. While AEC was collected longitudinally, it was still regressed over an indicator variable for ETI initiation because time since initiation was not meaningful. The multivariable model adjusted for age, gender, CFTR mutation, initial disease severity level, *Pseudomonas* spp. colonization, MRSA colonization, other bacterial colonization, fungal colonization, and inhaled steroid use.

## Results

1. Baseline demographics and characteristics

Summaries of baseline demographics and patient characteristics are presented in Table 1. All but five patients with complete AEC data also had complete IgE data. The characteristics of the sample of patients in the IgE analysis were similar to the characteristics of the sample of patients in the AEC analysis.

2. IgE and ETI therapy

**Table 1:** Subject characteristics

Characteristics	All patients (n = 85)	IgE analysis (n = 80)	AEC analysis (n = 54)
Gender			
Male: n (%)	41 (48.2%)	38 (47.5%)	27 (50.0%)
Female: n (%)	44 (51.8%)	42 (52.5%)	27 (50.0%)
Age			
Between 18 and 30 years	38 (44.7%)	37 (46.3%)	23 (42.6%)
Between 30 and 50 years	36 (42.4%)	33 (41.2%)	24 (44.4%)
Above 50 years	11 (12.9%)	10 (12.5%)	7 (13.0%)
Mean (SD)	34.2 (10.7)	33.7 (10.3)	34.7 (11.0)
Median (IQR)	31.8 (12.8)	30.9 (13.0)	32.6 (13.2)
CFTR Mutation Information			
delF508 Homozygous	49 (57.7%)	48 (60.0%)	32 (59.3%)
delF508 Heterozygous	34 (40.0%)	30 (37.5%)	22 (40.7%)
Other Mutation	2 (2.4%)	2 (2.5%)	0 (0.0%)
Initial Disease Severity			
Normal (ppFEV1 > 90%)	23 (27.1%)	22 (27.5%)	13 (24.1%)
Mild (70% < ppFEV1 ≤ 90%),	26 (30.6%)	25 (31.2%)	19 (35.2%)
Moderate (40% < ppFEV1 ≤ 70%)	25 (29.4%)	23 (28.7%)	17 (31.5%)
Severe (ppFEV1 ≤ 40%)	10 (11.8%)	10 (12.5%)	4 (7.4%)
Not Available	1(1.2%)	0 (0.0%)	1 (1.9%)
Pre-ETI Inhaled Steroids			
Yes	51 (60.0%)	49 (61.3%)	30 (55.6%)
No	33 (38.8%)	30 (37.5%)	24 (44.4%)
Not Available	1 (1.2%)	1 (1.3%)	0 (0.0%)
Colonization			
MRSA	25 (29.4%)	21 (26.3%)	17 (31.5%)
*Pseudomonas*	50 (58.8%)	48 (60.0%)	29 (53.7%)
Other Bacteria	32 (37.6%)	31 (38.8%)	21 (38.9%)
Fungus	36 (42.4%)	36 (45.0%)	25 (46.3%)
Not Available	1 (1.2%)	1 (1.3%)	0 (0.0%)

“All patients” refers to all patients included either the IgE analysis, AEC analysis, or both

*IgE* Immunoglobulin E, *ETI* Elexacaftor/Tezacaftor/Ivacaftor, *ppFEV1* percent predicted Forced Expiratory Volume in 1 s, *MRSA Methicillin Resistant Staphylococcus Aureus, AEC* Absolute Eosinophil Count

**Fig. 1:**
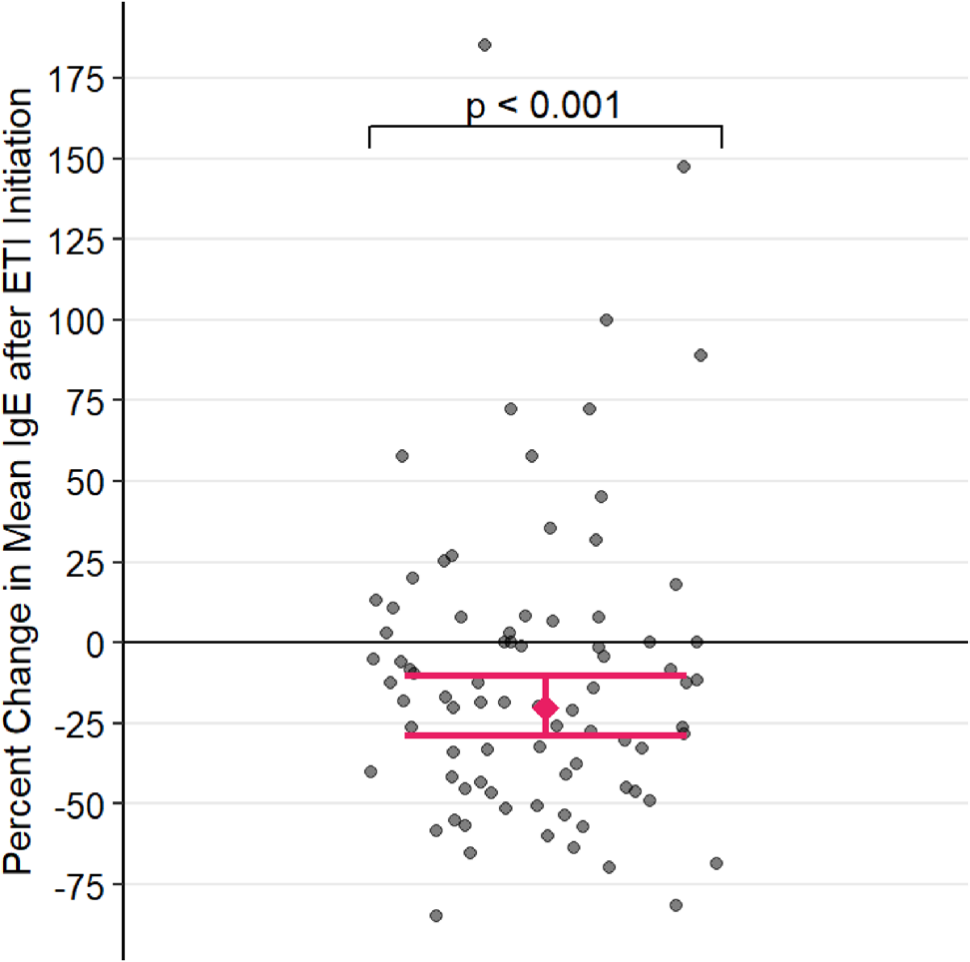
Percent change in mean total IgE = Immunoglobulin E after ETI = Elexacaftor/Tezacaftor/Ivacaftor initiation, with 95% confidence intervals computed from the multivariable mixed-effect model. Each point represents the percent change in 12-month mean IgE of a single patient after starting ETI

The estimated change in IgE after ETI initiation is plotted in Fig. 1. After adjusting for covariates, patients’ log-transformed 12-month mean total IgE decreased by 0.224 [p < 0.001, 95% CI (0.108, 0.341)], corresponding to a reduction of 20.10% [95% CI (10.23%, 28.89%)], following ETI initiation. Across the entire study (both before and after ETI), inhaled steroids were associated with 0.874 higher log mean IgE, [p = 0.023, 95% CI (13.20%, 406.85%)]. However, in the multivariate model inhaled steroids were not found to statistically significant associate with the magnitude of total IgE change pre and pos ETI initiation.

3. Post-ETI initiation change in IgE and lung function

After determining that ETI initiation was associated with a change in 12-month mean total IgE, we investigated whether initial lung function impacted the magnitude of the change in total IgE after ETI. In this mixed model, the reduction of total IgE following ETI initiation remained statistically significant (p < 0.001). However, total IgE at baseline did not significantly differ by initial disease severity (p = 0.206), and the reduction in total IgE following ETI therapy did not significantly differ by initial disease severity (p = 0.890). These results showed that in our patients, the magnitude of the change in IgE after ETI does not seem to depend on initial disease severity.

4. Post-ETI change in IgE and microbiological colonization

We also investigated whether fungus colonization, *Pseudomonas* colonization, or MRSA colonization alone modified the magnitude of the change in mean total IgE after ETI using subgroup analysis through univariate and multivariable models. Univariate and multivariable models produced similar results, so all values reported below are adjusted estimates.

**Fig. 2:**
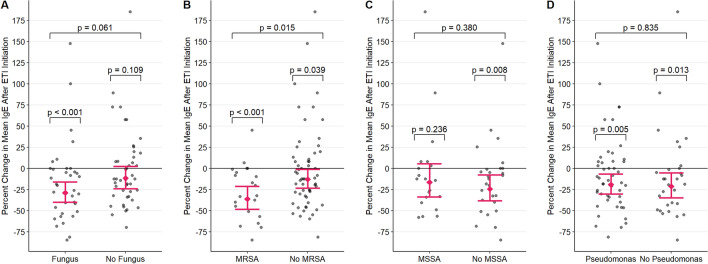
Percent change in mean total IgE = Immunoglobulin E after ETI = Elexacaftor/Tezacaftor/Ivacaftor initiation in the **A** presence of fungus or **B** presence of MRSA = Methicillin Resistant Staphylococcus Aureus or **C** presence of MSSA or **D** presence of Pseudomonas, with 95% confidence intervals from the multivariable mixed- effect model. Each point represents the percent change in 12-month mean IgE of a single patient after starting ETI. p-values above each group denote the significance of the percent change for that group from no change, and p-values connecting groups denote the significance of the difference in percent change across groups

As shown in Fig. 2A, after starting ETI, patients with at least one positive fungal culture prior to ETI experienced a significant percent reduction in mean total IgE [− 28.91%, p < 0.001, 95% CI (− 39.89%, − 15.93%)], but patients with no history of fungal colonization did not [− 11.68%, p = 0.109, 95% CI (− 24.17%, + 2.87%)]. The difference in the average magnitude of IgE change between patients with and without at least one positive fungal culture was not significant (p = 0.061). Figure 2B displays our results for MRSA. The percent reductions in mean total IgE following ETI initiation were significant in both patients with [− 36.26%, p < 0.001, 95% CI [− 48.49%, − 21.12%]) and without MRSA colonization [− 12.83%, p = 0.039, 95% CI (− 23.48%, − 0.69%)]. The magnitude of reduction was significantly larger in patients with MRSA colonization (p = 0.015). After starting ETI, a significant percent reduction in mean IgE was observed in patients without [− 24.48%, p = 0.013, 95% CI (− 38.28%, − 7.59%)] MSSA colonization but not in patients with [− 13.29%, p = 0.236, 95% CI (− 31.63%, + 9.98%)] MSSA (Fig. 2C). There was no significant difference in the average magnitude of IgE change between patients with and without MSSA colonization (p = 0.380). Finally, a significant percent reduction in mean total IgE was observed both in patients with [− 19.32%, p = 0.005, 95% CI (− 30.48%, -6.36%)] and without [− 21.33%, p = 0.013, 95% CI (− 34.80%, − 5.07%)] *Pseudomonas* colonization after starting ETI (Fig. 2C). There was no significant difference in the average magnitude of IgE change between patients with and without *Pseudomonas* colonization (p = 0.835).

In summary, these results indicate that without considering the effects of the presence of other microorganisms, a reduction in mean total IgE was observed in the presence and not absence of a positive fungal culture, and in the absence and not presence of MSSA. The presence of Pseudomonas did not impact the IgE decline post ETI. The magnitude of reduction in mean total IgE after ETI initiation was only impacted by prior MRSA colonization.

5. AEC and ETI

**Fig. 3:**
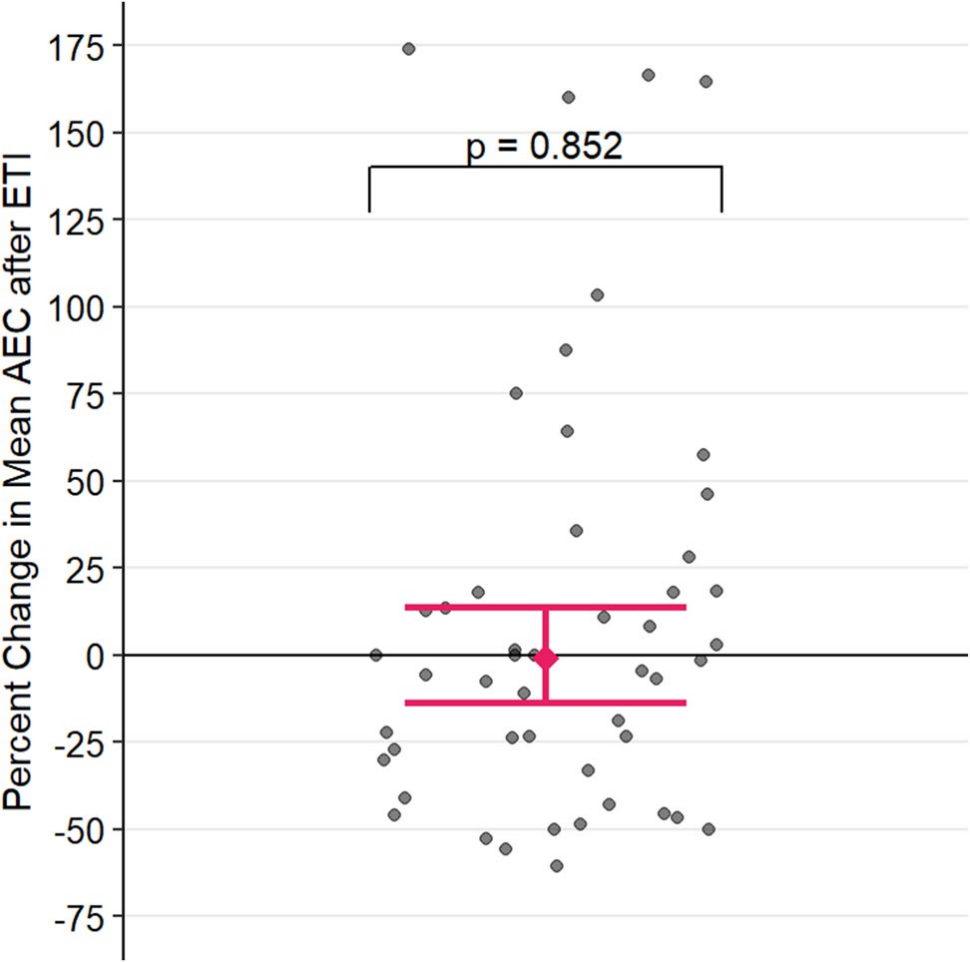
Association between *ETI* = Elexacaftor/Tezacaftor/Ivacaftor initiation and log *AEC* = Absolute Eosinophil Count, with 95% confidence intervals computed from the multivariable mixed-effect model. The p-value denotes the significance of change in mean log AEC after starting ETI. Each point represents one observation, and patients can contribute multiple observations. Circles represent observations from female patients, while triangles represent observations from male patients

The estimated percentage change in AEC after ETI initiation is plotted in Fig. 3. Without controlling for any covariates, ETI initiation was not associated with a significant change in AEC [p = 0.911, 95% CI (− 0.144, 0.128)]. The multivariable model showed that over the course of the entire study, men have statistically significantly lower AEC than women. On average, men had 37.71% lower AEC than women [p = 0.009, 95% CI (12.18%, 55.81%)]. However, even after adjusting for covariates, ETI initiation still had no association with a change in log-transformed AEC [p = 0.851, 95% CI (− 0.149, 0.124)].

## Discussion

A large subset of patients with CF develop evidence of T2 inflammation as demonstrated by the presence of blood and airway eosinophilia, robust elevations in their total IgE, and evidence of allergen sensitization [3–12]. When present, this T2 inflammatory state contributes to the severity of disease and development of disease exacerbations [11, 12] as can be demonstrated by clinical improvements observed with institution of biologics that target the T2 mediator IL-5. [13]

The treatment of patients with CF has been revolutionized by the efficacy of CFTR modulator therapy. The mechanism behind the improvement in patient outcomes is a continued topic of investigation as our understanding of the role of CFTR function evolves. It is unclear whether institution of CFTR modulator therapy will impact a previously established T2 inflammatory state.

To our surprise, multivariable analysis of AEC data in this study, after excluding measurements obtained during exacerbation and/or IV antibiotic use, demonstrated no change following therapy initiation. Eosinophilia is the quintessential defining feature of T2 inflammation [20, 21]. As such the absence of a change in AEC signifies that initiation of CFTR modulator therapy had no impact on these central defining components of T2 inflammation. This clinically would lead to no impact on the CFAOS presentation and CF exacerbations related to T2 inflammation in PwC and an eosinophilic phenotype.

In contrast to AEC, however, this data demonstrated a ~ 20% reduction in 12-month mean total IgE following ETI triple therapy initiation after controlling for multiple factors including demographic data and inhaled steroid use in the absence of an apparent impact on the T2 signature, this impact on total IgE begs an alternative explanation. CFTR dysfunction results in impaired mucus production and clearance, thus promoting microbial colonization in the airway. These microbes and, in particular, fungi, can function as allergens, especially in the presence of a T2^high^ state and it is well recognized that CF patients frequently display aspergillus-specific IgE and, indeed, this disease is often complicated by allergic bronchopulmonary aspergillosis [22–24]. The CF airway is also frequently contaminated with *Staphylococci.* While not an allergen, *staph* are copious producers of numerous superantigens that non-specifically (that is, in a T cell receptor-independent fashion) can broadly activate numerous families of T lymphocytes. This study showed that the total IgE declined post ETI in subjects previously colonized with MRSA, Pseudomonas and fungi. MSSA colonization was associated with no change in IgE pre and post ETI, while in the absence of MSSA, the IgE declined post ETI. CF has been associated with high expression of staph-derived superantigens in the airway [25, 26]. Insofar as many of these Th2 lymphocytes, staph superantigens can drive a “surge” of Th2 cytokines, including especially, IL-4 and IL-13, cytokines responsible for IgE production [26]. Allergens fairly rapidly influence IgE levels as demonstrated by the surges in IgE observed during aeroallergen seasons in allergic rhinitis patients and rapid reduction at the end of the exposure; ETI improves mucociliary clearance, and reduces fungal, Pseudomonal and other bacteria related burden, thereby reducing IL-4/IL-13 production and subsequently IgE levels. In addition, *Staphylococcus aureus* can also cause epigenetic changes, DNA methylation leading to a persistent inflammatory state [27]. Immune factors leading to eosinophilia, including the constitutive production of IL-25/IL-33/TSLP by epigenetically modified airway epithelial cells may be less influenced by ETI and may remain constant. This hypothesis is supported by our data demonstrating a reduction in total IgE in patients with a history of fungal colonization but was not reduced in patients with no prior history of fungal colonization. Thus, reduction in antigen (fungal) and superantigen (*Staph*) load secondary to ETI therapy initiation could explain the reduced total IgE production in the absence of ETI therapy having an effect on ameliorating the underlying T2^high^ state.

This analysis serves as an important starting point to continue the evaluation of CFTR modulator therapy from an immunologic perspective. These results are intriguing and warrant further studies to elucidate the exact effect of ETI on T2 inflammation. Based on our current findings and previous studies that show a correlation between T2 inflammation based on AEC and IgE levels and frequency of exacerbations [12], we suspect the ETI may not influence the rate of exacerbation in a subgroup of people with CFAOS and an eosinophilic phenotype.

Further studies to elucidate the impacts of possible confounding variables in a larger patient population are warranted. Moreover, studies investigating additional markers of the T2 inflammatory response, especially those utilizing samples obtained from airway will prove valuable. Lastly, all of the patients included in the study initiated ETI therapy during adulthood. Given that early initiation of ETI during childhood is increasingly common, children may have had less infectious history, reduced antibiotic use, and less airway injury and thus may not have had an epigenetically established type 2 inflammatory signature prior to ETI initiation. Further studies analyzing the impact of ETI on T2 inflammatory markers in children could therefore be valuable in investigating the role of CFTR modulator therapy on T2 inflammation. MSSA colonization occurs usually early in life, while Pseudomonas, fungus and MRSA occur later. This may possibly lead to epigenetic changes in airway inflammation related to MSSA colonization and persistent T2 inflammation in this subpopulation.

There are important considerations and limitations regarding this study. There are inherent limitations in performing a retrospective analysis due to limited data measurement frequency, varying frequency of clinical assessment of exacerbation status, and possible unknown microbiological colonization history and allergen sensitization prior to subject care establishment at our center. As per standard of care, at our program, when the sputum provided is an adequate amount, fungal, bacterial and AFB cultures are performed from the same specimen. When the amount is small, only bacterial cultures are performed, and when the patient cannot produce sputum, throat swabs are performed. Based on registry data, the ETI lead to a decrease in bacterial and fungal colonization, but in the absence of adequate sputum or bronchoalveolar lavage specimen, this is hard to prove. A prospective study of influence of bacterial and fungal colonization on T-2 inflammation post ETI may be informative. T2 inflammatory markers are impacted by numerous confounders including exposure to other allergens, systemic and topical corticosteroid use, and others. A future prospective study is essential using more robust markers of T2 inflammation including serum cytokines, periostin, and fractional exhaled nitric oxide. Retrospective collection of systemic steroid bursts used in the study group was not possible.

## Conclusion

This study demonstrates that there is a statistically significant percent reduction in mean total IgE but no change in AEC following ETI initiation. ETI may lead to decreased antigen and superantigen load in the airway as a result of improved mucociliary clearance and these changes may drive the decline in total IgE, without influencing the epigenetic drivers of eosinophilic inflammation. Further studies are warranted to determine the underlying mechanism of ETI impact on T2 inflammation and possible role for asthma immunomodulator therapy post ETI initiation in CFAOS.

## Data Availability

The datasets generated and/or analysed during the current study are not publicly available due to possibly leading to subject identification, (single center, and rare disease subjects) but the statistical analysis is available from the corresponding author on reasonable request.

## Abbreviations

AEC: Absolute Eosinophil Count
CF: Cystic fibrosis
CFAOS: CF-asthma overlap syndrome
CFTR: Cystic Fibrosis Transmembrane Regulator
ETI: Elexacaftor/Tezacaftor/Ivacaftor
IgE: Immunoglobulin E
IL: Interleukin
TSLP: Thymic stromal lymphopoietin
ppFEV1: Percent predicted forced expiratory volume in 1 s
MRSA: Methicillin resistant *Staphylococcus Aureus*
